# Can Teledentistry Improve the Monitoring of Patients during the Covid-19 Dissemination? A Descriptive Pilot Study

**DOI:** 10.3390/ijerph17103399

**Published:** 2020-05-13

**Authors:** Amerigo Giudice, Selene Barone, Danila Muraca, Fiorella Averta, Federica Diodati, Alessandro Antonelli, Leonzio Fortunato

**Affiliations:** 1 School of Dentistry, Department of Health Sciences, Magna Graecia University of Catanzaro, 88100 Catanzaro, Italy; danila.muraca@studenti.unicz.it (D.M.); fiore.averta@gmail.com (F.A.); federica.diodati@studenti.unicz.it (F.D.); antonellicz@gmail.com (A.A.); leo@unicz.it (L.F.); 2 Unit of Dentistry, “Mater Domini” Hospital, COVID-19 Reference Hospital for Calabria Region, 88100 Catanzaro, Italy

**Keywords:** telemedicine, Covid-19, dentistry, WhatsApp, teledentistry, dental public health, community dentistry

## Abstract

The aim of this pilot study was to describe the advantages of telemedicine (TM) in dental practice during the current national emergency condition due to the Covid-19 dissemination. At Department of Oral Surgery and Pathology—Magna Graecia University of Catanzaro, regional reference center for Covid-19—two groups of patients were determined: patients with urgent conditions (group U) and patients in follow-up (group F). Both groups were instructed to implement remote consultations using a messaging service (WhatsApp Messenger, WhatsApp Inc., Mountain View, California, USA) to send photos. A total of 418 photos were collected by 57 patients. Thirty-four photos were obtained by five patients in the U group after surgical procedures. All patients sent photos on the established evening, except for two patients who sent two photos outside the set days. In the F group, 384 photos were collected by 52 patients. None of them sent more photos than the number that was established by the protocol. Telemedicine allowed a monitoring of all patients, reducing costs and limiting human contact, decreasing the risk of Covid-19 dissemination.

## 1. Introduction

In Wuhan, China, an unusual pneumonia appeared in December 2019. Because a new Coronavirus was the etiological factor, it was renamed “Coronavirus disease 2019” (Covid-19) by the World Health Organization (WHO) [1]. A rapid spread of Covid-19 occurred across both China and the world, defining this pathological condition as a pandemic. In Europe, Italy became one of the first countries to be severely affected, with a rapidly increasing number of infected patients. With the decree of 9 March 2020, Italy was declared a “red zone” by the government with restrictions on travel and social meetings [2]. In particular, in some cities, obligatory quarantine was imposed, preventing any type of movement. In this emergency situation, new models of assistance were encouraged, limiting the direct contact between doctor and patient. When possible, it is recommended to avoid hospitals, dental offices or other medical offices because they are health facilities recording an increased risk of cross infection [3]. Specifically, the route of transmission of this Coronavirus has a significant involvement of dental practice. Many dental procedures produce aerosols and droplets contaminated by microorganisms that lead to an easier spread of infections [4].

To observe government decisions, the Internet is to date the only way to build a significant linker platform for all medical professionals and particularly for dental practitioners. Communication technologies, such as smartphones, tablets and laptops, supported the rapid development of telemedicine (TM) as a new concept of healthcare to deliver care across distances [5]. TM completely modified the traditional medical approach of working, promoting a virtual method of visits, consultations, and follow-up instead of physical contact and face-to-face clinical evaluations. Although existing since 1969, in Italy, TM and teledentistry are now largely appreciated in minimizing the risk of increased Covid-19 dissemination [6]. The most significant advantages of TM are: (1) real-time consultation and (2) store and forward data; however, TM is not free from disadvantages, including the exchange of sensitive information, the commitment to confidentially, the commitment to security and a large volume of data stored [7].

The Covid-19 pandemic has created unique challenges in ensuring healthcare. Nevertheless, the possibility of using telehealth systems and methodologies in dentistry, defined as teledentistry, could improve the quality and efficiency of dental health care [8]. For this reason, the aim of this pilot study was to explore, in the regional reference center for Covid-19, the role of teledentistry for the follow-up management of patients who have undergone urgent surgical treatments.

## 2. Materials and Methods

A pilot study was conducted from 20 February until 20 March 2020 at the department of Health Science of Oral Surgery and Pathology of Magna Graecia University of Catanzaro, Italy, that was designed to be the regional reference center for Covid-19. The following protocol was conducted in accordance with the “Ethical Principles for Medical Research Involving Human Subjects” statement of the Helsinki Declaration. We proposed our study protocol to the Ethical Committee of Magna Graecia University however, in this emergency period, it was not possible to receive a quick answer and a protocol number. All patients gave their informed consent to their participation in the study and the storage of their data.

### 2.1. Study Sample

All participants deliberately gave their consent to take part in the study. The inclusion criteria of the study sample were the following: (1) 18 years of age or over; (2) completed the Covid-19 questionnaire [9]; (3) a negative previous pathological and pharmacological history; (4) possession of a smartphone with the following minimum requirements: 5 Megapixel front integrated camera and 8 Megapixel principal integrated camera, installation of the WhatsApp application, and access to an Internet network. The exclusion criteria included: (1) patients with claimed symptoms of Covid-19; (2) patients with urgent or post-op complications that should come back to the clinic [10,11,12,13]; (3) patients or relatives with motor difficulties of any nature that prevented the correct capture of images.

### 2.2. Study Protocol

The study sample included 2 different groups: patients with urgent pathologies (U group) and patients in follow-up (F group). The F group included: patients with chronic conditions (precancerous lesions, medication-related osteonecrosis of the jaw (MRONJ), autoimmune diseases) (FC group); patients in follow-up after surgical treatments performed some weeks before the 20th February 2020 (SF group) because they needed a clinical follow-up in the first period of the Covid-19 pandemic. During this month, patients requiring the first remote medical evaluation were distinguished in U or F group according to their clinical condition. The remote visit was preferably performed by making a group videocall between the patient and a General Practitioner (GP). If a GP was not available, a video consultation was performed alone with the patient. Before starting the consultation, patients had to show their completed Covid-19 questionnaire and good-quality pictures. Medication lists and radiographs were also required. If patients required it, an electronic medical prescription could be sent to them at any time. If complications occurred in either of the groups, remote consultations should have been substituted for the inspection itself.

For all patients, an evaluation of the adherence to the protocol was performed using established remote consultations as an electronic monitoring method. Adherence was defined in terms of precision in respect of the time and methods of sending photos. Adherence monitoring (AM) classified each patient into one of the following values: (1) AM = 0 if the patient showed a reduced adherence to the protocol; (2) AM = 1 if the patient showed a good adherence to the protocol; (3) AM = 2 if the patient showed an increased adherence to the protocol.

### 2.3. U Group

In case of urgent not-deferable surgical procedures, all subjects received a surgical treatment as atraumatic as possible [14]. All therapies were carried out under antibiotic therapy according to the pathological condition and after disinfection of the oral cavity with chlorhexidine 0.20%. In all cases, stitches were applied with single knots using 4/0 absorbable suture. All patients were given a reminder sheet containing instructions to follow in regard to antibiotics, pain-relievers and anti-edema therapy, as well as instructions for how to take and send the photographs. The post-op photos had to be sent on the evenings of the 3rd, 7th and 14th post-operative days. Each patient was given a paper ruler and instructed to use it to measure the interincisal distance at maximum opening.

### 2.4. F Group

In cases of follow-up for patients with chronic pathological conditions (FC group) or who have undergone previous surgical treatments (FS group), all the instructions for therapy, and for taking and sending the photographs were explained in detail by phone. Precancerous lesions were analyzed in detail to assess any clinical modification of the tissue. For the autoimmune pathologic conditions, recurrence, pain and functional limitations were eventually evaluated. In MRONJ patients, the size and stage of the lesion, pain, presence of infection and eventual recurrence were determined. Photos had to be sent every week.

### 2.5. Data Collection Method

Photos had to be taken by the patient themselves or with the help of a direct relative. Patients had to use the camera of their smartphone to capture only the following images: (1) a picture of the surgical site; (2) a picture of the face; (3) a picture of the maximum buccal opening with a visible ruler after surgical treatment of hard tissues [15]. A self-assessment questionnaire on pain levels according to the Visual Analog Scale (VAS) was required for surgical patients. These data had to be sent from the examiner’s smartphone using a messaging service [16]. The WhatsApp application uses end-to-end encryption, so the communication between the sender’s phone and the receiver’s phone is secure. In addition, the telephone to which the photos were sent was accessible only to a restricted group of professionals through the use of specific authentication credentials. All data were then stored on special hard disks in full compliance with the General Data Protection Regulation (GDPR) in force since May 4, 2016.

Each patient could request information for any needs at any time: a telephone number was provided that had been set up precisely for this purpose, as well as for data collection. The smartphone used to receive photos was an iPhone 7 (Retina HD, 4.7” widescreen Multi Touch LCD, Apple Inc, Cupertino, Calif).

### 2.6. Data Analysis

Recorded data was transferred to an EXCEL file. Descriptive statistical analyses were performed using frequencies and percentages for categorical data and mean and standard deviations for continuous data.

## 3. Results

### 3.1. Study Sample

From 20 February 2020 to 20 March 2020, 57 patients participated in the study. All data are reported in Table 1. The study sample included 35 women (61.4%) and 22 men (38.6%), with a mean age of 43.8 ± 15.7 years. A sample size calculation was not performed for our study; during the Covid-19 pandemic all patients with the inclusion criteria were enrolled in this study.

Participants were distinguished by their province of residence: 12 (21%) lived in the province of Catanzaro; 21 (36.8%) in Cosenza; 5 (8.8%) in Crotone; 5 (8.8%) in Vibo Valentia; 14 (24.6%) in Reggio Calabria (Table 1). In particular, seven patients included in the study were residents of towns subject to further restrictions for the containment of the virus which prohibited circulation outside the respective municipality such as Paola (CS) or Montebello Jonico (RC).

### 3.2. U Group

**Figure 1:**
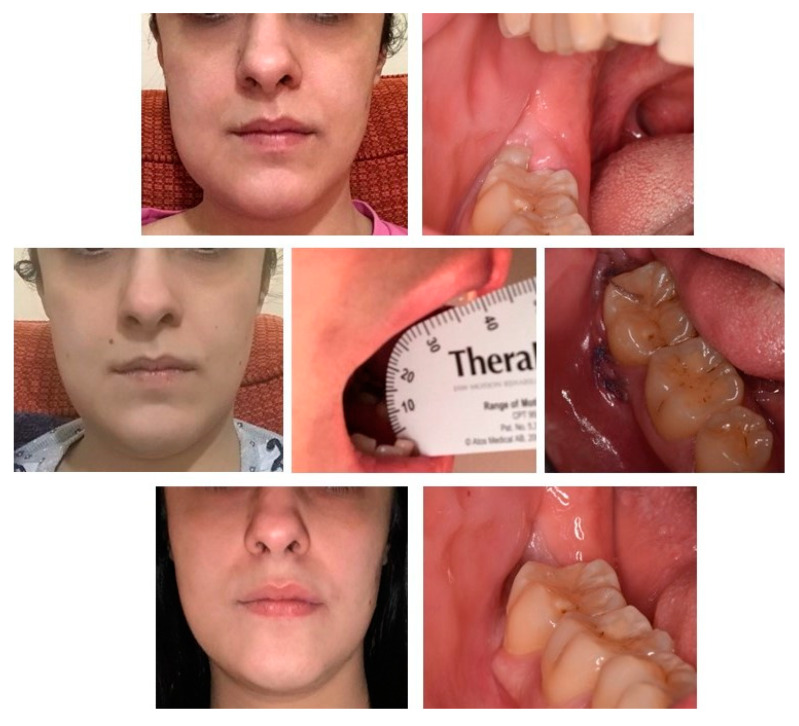
A female patient, 28 years old, undergoing a periodic follow-up after a surgical treatment of third molar removal. The collection of photos highlighted the ease of taking good quality photos and examining them, allowing management through telemedicine.

Data are showed in Table 2. Out of 5 (8.8%) patients who had to go to the clinic for urgent pathologies, 2 have received a surgical treatment for a dental abscess (Figure 1), 1 for a neoplastic lesion, 1 for a dental fracture and 1 for an oroantral communication. 

A total of 34 photos were collected from patients belonging to the U group. Of these, 21 were for dental abscesses, 4 were for dental fractures, 5 were for oroantral communication, and 4 were for neoplastic lesions. All patients sent photos on the evening of the 3rd, 7th and 14th post-operative day, but two patients sent two photos outside the set days. One of these had swelling and felt pain, the other was afraid of a slight bleeding. The mean VAS pain score was 8 on the 3rd day, 6 on the 7th day and 4 on the 14th day. The average value of the interincisal distance at maximum opening was 30 mm on the 3rd day, 41 mm on the 7th day and 42.5 mm on the 14th day.

### 3.3. F Group

**Figure 2:**
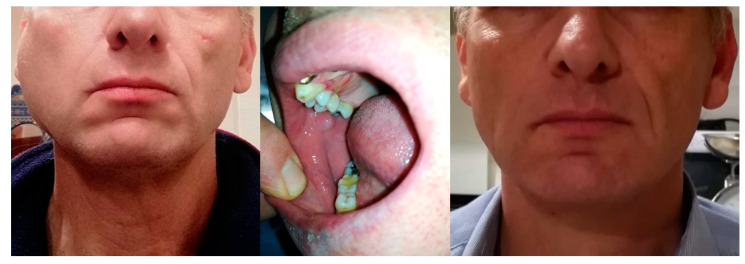
A male patient, 55 years old, undergoing a periodic follow-up after the telematic first visit. A diagnosis of sialolithiasis was performed by analyzing medical history and patient photos. Behavioural instructions and pharmacological therapy were given to the patient. Photo collection showed facial swelling in the right parotid region and intraoral condition with swelling of the Stenone duct at the first consultation; face healing occurred after two weeks.

Data are showed in Table 2. The follow-up group included 52 patients (91.2%): 24 (46.2%) were followed for chronic pathologies (FC group), 17 (32.7%) were in post-operative follow-up (FS group) and the remaining 11 patients (21.1%) were included after the first telemedical consultation. The FC group included 7 patients with precancerous lesions, 4 with autoimmune diseases and 13 with MRONJ. The FS group included 7 patients that had undergone third molar surgery, 4 patients were followed after a cystic enucleation and 6 after a biopsy of suspected oral lesions. Eleven patients who required the first visit showed the following deferable conditions: mycotic infection (2); traumatic ulcer (2); sialolithiasis (1) (Figure 2); third molar pericoronitis (5); and burning mouth syndrome (1).

A total of 384 photos were collected. In the FC group, 216 photos were stored, most of which concerned patients following MRONJ (117) and precancerous lesions (63) (Table 2). For precancerous lesions, no clinical tissue alteration was detected. In patients with MRONJ, bone sequestrum did not show particular modifications: no superinfection or stage worsening were observed.

In the FS group, 153 photos were collected: 63 by patients after third molar surgery, 54 by patients undergoing biopsy and 36 by patients undergoing cystic enucleation. Finally, of the 384 photos, the remaining 15 showed the follow-up of the group of patients who underwent the first telemedical consultation.

None of the F group subjects sent more photos than those established by the protocol.

#### Adherence to the Protocol

In the U group, 3 patients (5.2%) showed AM = 2, while 2 patients had AM = 1 (3.5%). In the F group, 4 patients showed AM = 0 (7.01%), 1 patient showed AM = 1 (1.7%) and 47 patients had AM = 1 (82.4%).

## 4. Discussion

The aim of this study was to highlight the usefulness of teledentistry during a particular historical period in Italy. Because our dental clinic is the regional reference center for Covid-19, the typical clinical procedures were reserved for urgent cases only, implementing an alternative means of routine evaluation to manage patients in follow-up.

As carried out by the Chinese government, the Italian government also had to adopt significant restrictions on people, imposing on them to stay at home and to limit their social lives [2]. While going out is a ban for any but urgent reasons, in Italy TM is playing a key role in reducing the risk of Covid-19 dissemination. The increasing availability of the Internet and the development of powerful and versatile devices such as smartphones, tablets and laptops could, in the future, allow TM to become relevant in today’s society by changing the way in which care services and healthcare are provided worldwide [17]. Online conversations allow the exchange of several types of data: written or voice messages for diagnostic doubts and therapeutic suggestions, video messages for a better evaluation of a patient’s requirements and descriptions of problems [18]. Surely, high-quality images are the most common means of communication in TM, showing clinical examination reports, radiological investigation reports or simple photos of lesions [6]. Remote consultations could be performed either among medical professionals or between doctor and patients. During this historical period in Italy, patients could avoid travelling to the dental clinic without an effective indication, limiting the human contact between both doctor and patient and among patients in waiting rooms.

In this emergency month, photographic teleconsultations were performed both for first visits and for follow-up evaluations, ensuring a good distant management of the patient. After an adequate anamnesis in videocall and photographic assessments, many patients who required the first visit were managed with a remote follow-up which considered their deferable pathologies: mycotic infections, burning mouth syndrome, sialolithiasis, traumatic ulcers and third molar pericoronitis.

Aware that oral pathology must be based on clinical examination, in this particular month, TM was allowed to distinguish potentially malignant lesions from those that were really malignant and required an immediate approach, without the presumption of making a precise telediagnosis [19,20]. We were able to maintain under control patients with precancerous lesions, MRONJ and autoimmune diseases, comparing photographs received with the last photos performed at the dental clinic. For precancerous lesions, any clinical modifications were assessed to determine the risk of malignant transformation. Moreover, autoimmune diseases require the management of possible recurrence, pain and oral functional limitation [21,22]. For MRONJ, bone sequestrum, eventual superinfection and pain must be evaluated to control stability or a worsening of the lesions [23,24,25].

Furthermore, as reported by Khan, oral surgery could also benefit from teledentistry, not in terms of dental procedures, but as a means to monitor post-operative conditions [26]. After immediate surgical treatments, remote contact with a specialist allowed us to record a decreased pain score during post-operative evaluations associated with a good functional recovery without the need to return to our dental clinic. Thanks to the photo collection, we were able to analyze the surgical site, the concomitant oral hygiene and the improvement of oral functions.

In this study, teledentistry appeared to be a promising tool in the remote management of surgical and non-surgical patients, especially reducing costs and waiting times. Our patients came from different provinces of Calabria with a mean distance of 50 min by car from our dental clinic. Furthermore, seven patients lived in countries where severe restrictions were adopted to strengthen the containment of Covid-19. Without digitalization and an online conversation, the imposed social isolation would not have allowed follow-up of their clinical development.

Even though there are studies that support the use of telemedicine and WhatsApp in dental practice, the proposed study is completely original [16,27]. The remote follow-up and post-operative evaluation made clear any perplexity the patients had by giving instructions with a text message at any time. Two patients that had undergone urgent surgical treatments contacted us outside of the established days because they were concerned about swallowing, pain and bleeding. However, in the majority of the cases, there was no need for any real medical intervention.

The study limitations were the lack of control group and the sample size. Despite the limitations of analyzing photos taken by laypeople, TM allowed us to maintain a long-distance link with those patients who need periodical consultations [28].

If telemedicine is not error-free regarding its medicolegal implications, its indisputable advantages could provide a higher quality of assistance by breaking down space barriers, exchanging opinions and providing patients with the opportunity to ask for advice on diagnoses and treatments [6,7,8,9,10,11,12,13,14,15,16,17,18,19,20,21,22,23,24,25,26,27,28,29]. The optimization of software and procedures, especially for emergency situations, would lead to an improved management of patients, enhancing their quality of life. Although technology is of great support in the management of patients, especially in emergency periods, it is not devoid of disadvantages like costs, security and implications for the confidentially of data [7].

## 5. Conclusions

In this situation of government limitations, the awareness of being constantly monitored and the sensation of participating personally in the healing process thanks to teleconsultations, has helped to both increase patient compliance and establish a stronger doctor–patient relationship.

## Figures and Tables

**Table 1:** Data of the study sample.

Demographic Variables	Patients
Study sample	57
Sex	
Male	22 (38.6%) ^a^
Female	35 (61.4%) ^a^
Age groups	14 (24.6%) ^a^
≤34 yrs	23 (40.4%) ^a^
35–44 yrs	20 (35%) ^a^
>45 yrs	
Province of residence	
Catanzaro	12 (21%) ^a^
Cosenza	21 (36.8%) ^a^
Crotone	5 (8.8%) ^a^
Vibo Valentia	5 (8.8%) ^a^
Reggio Calabria	14 (24.6%) ^a^

^a^ Sample size of patients (percentage—%).

**Table 2:** Patients’ distribution and photo collection for each teleconsultation.

Study Group	Patients	Collected Photos
Study sample	57	418
*Urgent group (U group)*	5 (8.8%) ^a^	34
Dental abscess	2	21
Neoplastic lesion	1	4
Dental fracture	1	4
Oroantral communication	1	5
	52 (91.2%) ^a^	384
	11 (21.1%) ^a^	15
*Follow-up group (F group*)	5	6
(1) First medical evaluation	2	4
Third molar pericoronitis	2	2
Mycotic infection	1	3
Traumatic ulcers	1	0
Sialolithiasis		
Burning mouth syndrome (BMS)		
(2) Post-operative follow-up	17 (32.7%) ^a^	153
Third molar surgery	7	63
Cist enucleation	4	36
Biopsy	6	54
(3) Oral pathology follow-up	24 (46.2%) ^a^	216
Precancerous lesion	7	63
Autoimmune diseases	4	36
MRONJ	13	117

^a^ Sample size of photos collected (percentage—%).

## Abbreviations

Covid-19	Coronavirus disease 2019
TM	Telemedicine
UG	Urgent pathologies group
FC	Followed for chronic pathologies
FS	Post-surgical treatment follow-up
MRONJ	Medication-related osteonecrosis of the jaw
GP	General practitioner
